# Importance of the Global Regulators *Agr* and *SaeRS* in the Pathogenesis of CA-MRSA USA300 Infection

**DOI:** 10.1371/journal.pone.0015177

**Published:** 2010-12-02

**Authors:** Christopher P. Montgomery, Susan Boyle-Vavra, Robert S. Daum

**Affiliations:** 1 Section of Critical Care, Department of Pediatrics, University of Chicago, Chicago, Illinois, United States of America; 2 Section of Infectious Diseases, Department of Pediatrics, University of Chicago, Chicago, Illinois, United States of America; National Institute of Allergy and Infectious Diseases, National Institutes of Health, United States of America

## Abstract

CA-MRSA infection, driven by the emergence of the USA300 genetic background, has become epidemic in the United States. USA300 isolates are hypervirulent, compared with other CA- and HA-MRSA strains, in experimental models of necrotizing pneumonia and skin infection. Interestingly, USA300 isolates also have increased expression of core genomic global regulatory and virulence factor genes, including *agr* and *saeRS*. To test the hypothesis that *agr* and *saeRS* promote the observed hypervirulent phenotype of USA300, isogenic deletion mutants of each were constructed in USA300. The effects of gene deletion on expression and protein abundance of selected downstream virulence genes were assessed by semiquantitative real-time reverse-transcriptase PCR (qRT-PCR) and western blot, respectively. The effects of gene deletion were also assessed in mouse models of necrotizing pneumonia and skin infection. Deletion of *saeRS*, and, to a lesser extent, *agr*, resulted in attenuated expression of the genes encoding α-hemolysin (*hla*) and the Panton-Valentine leukocidin (*lukSF-PV*). Despite the differences in *hla* transcription, the toxin was undetectable in culture supernatants of either of the deletion mutants. Deletion of *agr*, but not *saeRS*, markedly increased the expression of the gene encoding protein A (*spa*), which correlated with increased protein abundance. Each deletion mutant demonstrated significant attenuation of virulence, compared with wild-type USA300, in mouse models of necrotizing pneumonia and skin infection. We conclude that *agr* and *saeRS* each independently contribute to the remarkable virulence of USA300, likely by means of their effects on expression of secreted toxins.

## Introduction

Infections caused by methicillin-resistant *Staphylococcus aureus* (MRSA) are common and frequently severe [1], [2]. In the last decade, an increasing percentage of these infections have occurred among previously healthy individuals without traditional risk factors, including children [3]. Among these community-associated MRSA (CA-MRSA) infections, uncomplicated skin and soft tissue infections predominate; however, serious disease, including complicated skin and soft tissue infections and necrotizing pneumonia requiring hospitalization, also occur frequently [1]. The first reports of severe CA-MRSA infections in the United States implicated the genetic background USA400, as defined by pulsed-field gel electrophoresis, as the predominant cause [4], [5]. However, USA400 has essentially disappeared from the U.S. and has been replaced by USA300 [1], [6]. The reasons for the dominance of USA300 are not known, but some have interpreted the success as evidence for a fitness advantage conferred by the USA300 background. In support of this, comparative studies in several animal models of *S. aureus* disease have demonstrated that USA300 isolates are hypervirulent, when compared with USA400 [7] or selected health-care associated MRSA strains [8]. Although the relationship between fitness and virulence is a subject of ongoing discussion, understanding the molecular mechanisms of the extraordinary virulence of USA300 may provide insight into the pathophysiology of this remarkable genetic background.

In vitro and in vivo studies have uncovered unique molecular features of USA300 that may contribute to virulence. Genome sequencing has identified multiple mobile elements containing putative virulence genes, including enterotoxins (designated *sek2* and *seq2*), the arginine catabolic mobile element (ACME), and the Panton-Valentine leukocidin (PVL) [9], [10]. It is tempting to speculate that the CA-MRSA epidemic, and by extension the success of USA300, has been driven by acquisition of one or more of these “novel” virulence determinants. However, although ACME may enhance fitness [11], it does not enhance virulence in rodent models of infection [12]. Similarly, there is a strong epidemiologic association of PVL with the so-called CA-MRSA isolates, but a role for PVL in the pathogenesis of invasive disease has remained elusive [8], [13], [14], [15], [16], [17], [18], [19], [20].

An alternative explanation for the remarkable virulence of USA300 strains is that they have increased transcription (compared with USA400) of multiple core genomic global regulatory and downstream genes thought to be important in the virulence of *S. aureus* [7]. Among the genes encoding virulence factors that are upregulated in USA300 are *lukSF-PV* (encoding PVL) and *hla* (encoding α-hemolysin). *Hla* is known to promote virulence in animal models of necrotizing pneumonia and skin infection [14], [21]. The expression of *hla* and *lukSF-PV* is controlled in a growth-phase dependent manner by a number of global regulatory systems, including the accessory gene regulator (*agr*) and *S. aureus* accessory element (*sae*) operons, each of which is also upregulated in USA300 isolates [7], [22].

The *agr* operon consists of divergent transcripts, RNAII and RNAIII, whose expression is driven by promoters designated P2 and P3, respectively [23]. RNAII encodes 4 genes, called *agrDBCA*. *AgrD* and *agrB* encode a quorum sensing system that results in the production of an autoinducing peptide (AIP) that positively regulates *agr* expression. *AgrC* and *agrA* encode a two-component sensor histidine kinase (*agrC*) and response regulator (*agrA*). In addition to containing the gene that encodes the delta-hemolysin, RNAIII itself increases the expression of multiple exoproteins, including hemolysins, PVL, and enterotoxins [24], [25], [26]. Conversely, RNAIII negatively regulates proteins thought to be important in adhesion, including fibronectin-binding proteins and protein A [27], [28].

The *sae* operon consists of four genes, designated *saePQRS*. *saeR* and *saeS* encode a two component regulatory system [29]. The roles of *saeP* and *saeQ* are less well defined; they likely act to modulate *saeRS* expression, but *saeP* may also have an independent regulatory function [30]. Like *agr*, *sae* is an important global regulator in *S. aureus*. *Sae* may be important in CA-MRSA host-pathogen interactions, as *saeR* and *saeS* were upregulated after phagocytosis of USA300 and USA400 strains by neutrophils [31]. Deletion of *saeR* and *saeS* resulted in decreased expression of many virulence genes, including hemolysins, leukocidins, and serine proteases [32].

Although *agr* and *sae* have been studied in other *S. aureus* genetic backgrounds, their roles in the virulence of USA300 are less well characterized. Recently, deletion of *saeRS* has been found to attenuate virulence of USA300 and USA400 in mouse models of bacteremia and skin infection [33], [34]. However, the relative contributions of *agr* and *sae* to the pathogenesis of necrotizing pneumonia caused by USA300 was not explored.

We hypothesized that the increased expression of *agr* and/or *sae* we documented contribute to the hypervirulent phenotype of USA300. To assess this, we constructed isogenic deletion mutants of *agr* and *saeRS* in USA300. Using these constructs, we evaluated the contribution of each to expression of downstream virulence genes and to virulence in mouse models of skin infection and necrotizing pneumonia.

## Methods

### Ethics Statement

This study was carried out in strict accordance with the recommendations in the Guide for the Care and Use of Laboratory Animals of the National Institutes of Health. All animal experiments were approved and supervised by the Institutional Animal Care and Use Committee at the University of Chicago (protocol # 71694).

### Construction of deletion mutants

#### Deletion of *agr* (Table 1)

Bacteriophage φ11, provided by Jean Lee, was propagated in strain RN4220. Using standard methods, φ11 was used to transduce *agr::tet* from RN6911 (Richard Novick) to 923 (USA300 clinical isolate, soft tissue infection) [7], [24], [35]. In this construct, the entire *agr* locus, including *agrDBCA* and RNAIII, has been deleted [24]. The deletion of *agr* was confirmed by PCR and transcription analysis.

**Table 1:** Strains used in this study.

Strain	Description	Reference
Top10	*E. coli* cloning strain	
RN4220	*S. aureus* cloning strain	[24]
RN6911	*Agr* deletion mutant in RN6390	[24]
923	Wild-type USA300	[7], [35]
923*Δagr*	USA300 *agr* deletion mutant (*agr::tet*)	This work
923*Δsae*	USA300 *saeRS* deletion mutant (*saeRS::aad9*)	This work

#### Deletion of *saeRS* (Tables 1 and 2)

*saeRS* was deleted by allelic exchange using the pMAD vector, as described by Arnaud et al [36]. Briefly, fragments of DNA (500–600 bp) flanking the *saeR* and *saeS* genes of the *sae* operon were amplified from genomic DNA (strain 923) by PCR, restricted, purified, and cloned into pMAD flanking the *aad9* gene, that encodes spectinomycin resistance. The resulting plasmid was purified from *E. coli* and transformed into RN4220 by electroporation, with selection on medium containing spectinomycin (1000 µg/ml). The plasmid was purified from RN4220 and subsequently transformed into strain 923. Blue-white screening was used to identify mutants, as described [36]. Deletion was confirmed by PCR, sequencing and transcription analysis.

**Table 2:** Oligonucleotides used in this study.

Name	Sequence (5′-3′)	Application	Reference
RNAIII	F–TTCACTGTGTCGATAATCCA	RT-PCR	[49]
	R–GGAAGGAGTGATTTCAATGG		
	Probe–56-FAM/AAGATATCATTTCAACAATCAGTGACTTAGT/3IABlkFQ		
saeR	F–GTTGAACAACTGTCGTTTGATGA	RT-PCR	This work
	R–ACCACAATAACTCAAATTCCTTAATACG		
	Probe–56-FAM/ACTGTAAATGGTCACGAAGTCCCTATGC/36-TAMSp		
hla	F–CGGCACATTTGCACCAATAAGGC/FAM/G	RT-PCR	[7]
	R–GGTTTAGCCTGGCCTTCAGC		
lukF-PV	F–GCCAGTGTTATCCAGAGG	RT-PCR	[50]
	R–CTATCCAGTTGAAGTTGATCC		
	Probe–FAM/CGCGAAGAATTTATTGGTGTCCTATCTCGATCGCG/DABCYL		
spa	F–TTTGTCAGCAGTAGTGCCGTTTGC	RT-PCR	[51]
	R–GGCAACAAGCCTGGCAAAGAAGAT		
	Probe–56-FAM/CCAGGTTTAACGACATGTACTCCGTTACC/3IABlkFQ		
gyrB	F–AACGGACGTGGTATCCCAGTTGAT	RT-PCR	[51]
	R–TTGTATCCGCCACCGCCGAATTTA		
	Probe–56-FAM/AAATGGGACGTCCAGCTGTCGAAGTT/3IABlkFQ		
16S rRNA	F–TGGAGCATGTGGTTTAATTCGA	RT-PCR	[50]
	R–TGCGGGACTTAACCCAACA		
	Probe–/HEX/CGCTGACTTACCAAATCTTGACATCCTTCAGCG/DABCYL		
16S rRNA	F–CGGCCTAACTACGTGCCAGCAGC/JOE/G	RT-PCR	[7]
	R–GCGCTTTACGCCCAATAATTCC		
Sae.up	F–AAAGAATTCTTTTTTCACCTCTGTTCTTACGACC	*saeRS* deletion	This work
	R–AAAGGATCCCGCATTATGTTGCTTAATCTTATG		This work
Sae.down	F–AAACCATGGAAAACATTAAGCCATTTGTATTATA	*saeRS* deletion	This work
	R–AAAGAATTCAGACTAAAAAGAAGCTCCCA		This work
Aad9	F–AAAGAATTCATCGAATCCCTTCGTGAGCG	*saeRS* deletion	This work
	R–AAAGAATTCTAATAAACTATCGAAGGAAC		This work

Restriction sites are underlined.

### Bacterial Growth

For all experiments, bacteria were subcultured from frozen stocks onto tryptic soy agar (TSA) and incubated overnight at 37°C. The following afternoon, a single colony was inoculated into 5 ml of tryptic soy broth (TSB) and grown overnight (16 hours) at 37°C with shaking (250 rpm). The following morning, the overnight culture was diluted 1∶100 in fresh TSB (flask to volume ratio 5∶1). Bacteria were then harvested at the desired phase of growth, as assessed by OD_600_ and plating of serial dilutions.

### *In vitro* gene expression by semi-quantitative real-time reverse-transcription PCR (qRT-PCR)

#### RNA isolation and purification

At harvest (2, 3, or 6 hours), bacteria were pelleted by centrifugation. The supernatant was removed, and the pellet was resuspended in RNAprotect (Qiagen). Bacteria were again pelleted by centrifugation and were stored at −80°C. To isolate RNA, pellets were thawed on ice, resuspended in Trizol (Invitrogen), and lysed using glass beads (Lysing Matrix B) and the FastPrep system (Qbiogene). RNA was further purified using the RNeasy kit, including treatment with DNase (Qiagen). The quality and quantity of RNA were assessed by A_260_/A_280_ and by visualization on a 1.2% formaldehyde-agarose gel.

#### qRT-PCR

For each sample, 2 µg RNA was reverse transcribed using the High Capacity Archive cDNA Kit (Applied Biosystems). qRT-PCR was performed using luminescence upon extension (LUX™, Invitrogen) primers for *hla*, primers and molecular beacons (Invitrogen) for *lukF-PV*, and Prime Time™ qPCR primer probe mixes (Integrated DNA Technologies) for the genes encoding *saeR*, protein A (*spa*) and RNAIII. 16S rRNA was used as an endogenous control for *hla* and *lukF-PV*, and *gyrB* was used as an endogenous control for *saeR*, *spa* and RNAIII. Thermal cycling and detection was performed on an ABI7300 (Applied Biosystems). Standard curves were performed for each probe using serial dilutions of *S. aureus* genomic DNA. Relative quantification was calculated by the ΔΔC_T_ method, with expression of strain 923 at 2 hrs as the reference, as described [7].

### Assessment of protein abundance in culture supernatants by western blot

At harvest (2, 3, 4, or 6 hours), bacteria were pelleted by centrifugation and supernatants removed. Supernatants were then concentrated 10 fold and proteins separated by SDS-PAGE. Protein was transferred to a nitrocellulose membrane. After the membrane was blocked with skim milk/TBST, it was incubated with one of two primary antibodies, mouse anti-Spa or rabbit anti-Hla (Sigma). Following 3 washes with TBST, the membrane was incubated with secondary antibody conjugated to horseradish peroxidase (Sigma), washed, and incubated with a chemiluminescent substrate (Pierce). Membranes were visualized after exposure to film.

### Mouse model of necrotizing pneumonia

A mouse model of *S. aureus* pneumonia has been described [37]. Briefly, bacteria were grown to an OD_600_ of 3.0 (3 hrs 40 min) and pelleted by centrifugation (4000 g×15 min). The pellet was washed twice and resuspended in sterile PBS to achieve a concentration of either 3–4×10^8^ CFU (high inoculum) or 1×10^8^ CFU (low inoculum) per 20 µl. Six week old C57Bl/6 mice (Harlan) were sedated with intraperitoneal ketamine and xylazine, inoculated intranasally with 20 µl of bacteria (10 µl in each nare), held upright for 20 seconds to allow full aspiration, and returned to their cages. Animals were allowed to awaken, were given full access to food and water, and were observed at fixed intervals for signs of illness. Selected animals were removed 6 hours (high and low inoculum) or 24 hours (low inoculum) after inoculation and euthanized with pentobarbital. At sacrifice, the left lung was removed aseptically, placed in sterile PBS on ice, homogenized, and serial dilutions were plated on mannitol salt agar (MSA) for enumeration of *in vivo* bacterial survival. The right lung was removed, inflated with 1 ml of 10% neutral buffered formalin, and placed in formalin. The lung was then sequentially infiltrated with increasing concentrations of ethanol and xylene and embedded in paraffin. The tissues were sectioned and stained with hematoxylin and eosin. The severity of pulmonary pathology was assessed in a blinded fashion by a previously reported pulmonary severity score that included observations on extent and severity of pulmonary pathology, presence of bacterial colonies, and presence of necrosis [7]. Each section received a score from 0–11, with a score of ≥9 indicating severe necrotizing pneumonia.

### Mouse model of skin infection

Our mouse model of skin infection has been described [12]. Briefly, bacteria were grown as described above and resuspended to achieve a concentration of 1×10^7^ CFU/50 µl PBS. 6 week old Crl:SKH1 hairless immunocompetent mice (Charles River) were inoculated subcutaneously with 50 µl of *S. aureus* or PBS. Skin lesions were observed daily and dermonecrosis was measured. Randomly selected animals were euthanized 3 days after infection and skin lesions were excised, homogenized in sterile PBS, serially diluted, and plated on MSA.

### Data analysis

Expression by qRT-PCR and *in vivo* bacterial survival in lungs and skin lesions was compared by Student's t test. Histopathology severity scores were compared with the Mann-Whitney U test. Mortality rates in the pneumonia model and the rates of dermonecrosis were compared by the Fisher's exact test. Differences were considered statistically significant if p<0.05.

## Results

### Construction of mutants

923*Δagr* was resistant to tetracycline, as expected. PCR amplification demonstrated that the *agr* operon was absent. 923*Δsae* was resistant to spectinomycin and susceptible to erythromycin, confirming the loss of pMAD. PCR amplification using primers within the *saeRS* operon revealed absence of the genes, which was confirmed by DNA sequencing (data not shown). Deletion of *saeRS* or *agr* had no effect on growth, as assessed by OD_600_ and plating of serial dilutions at multiple time points during a growth curve experiment (data not shown).

### Deletion of *agr* or *saeRS* altered *in vitro S. aureus* gene expression

#### *Agr* (RNAIII)

**Figure 1:**
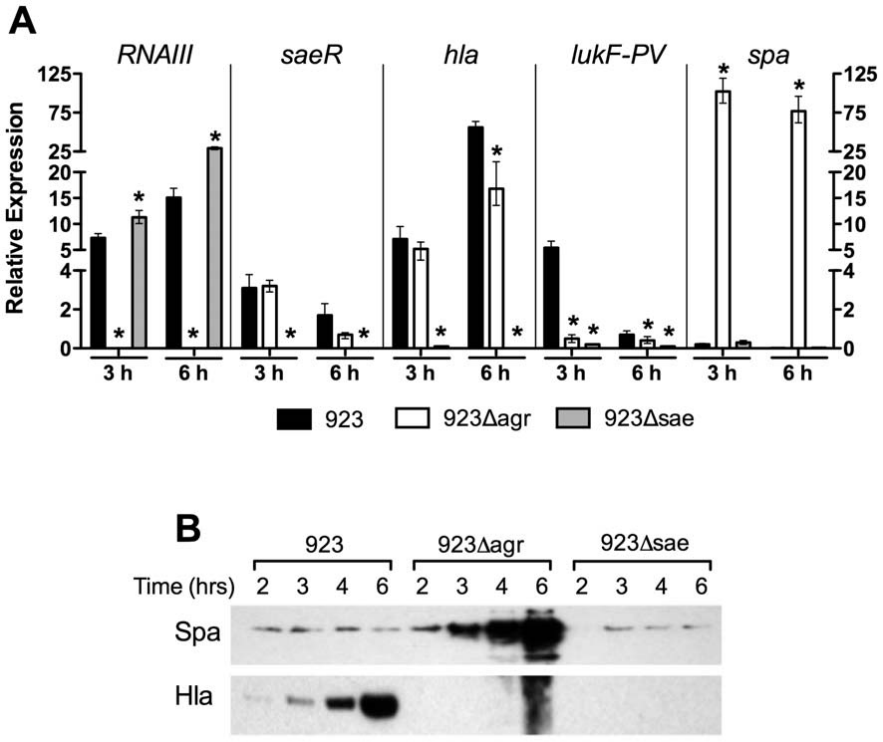
Deletion of *agr* or *saeRS* altered expression of global regulators (*RNAIII* and *saeR*) and downstream virulence factors (*hla*, *lukF-PV*, and *spa*). (A) Expression of global regulators and downstream virulence factors in strains 923, 923*Δagr*, and 923*Δsae* by qRT-PCR at 3 and 6 hrs of growth. Expression of each gene is quantified relative to expression in strain 923 at 2 hrs (corrected for housekeeping gene). Data are presented as mean ± SEM. * indicates p<0.05 compared with strain 923 at same time point. (B) Abundance of protein A (Spa) and α-hemolysin (Hla) in culture supernatants of strains 923, 923*Δagr*, and 923*Δsae* at 2, 3, 4, and 6 hrs of growth, as assessed by Western blot.

In strain 923, expression of RNAIII was highest at the post-exponential (6 hours) growth phase (Figure 1A). As expected, deletion of *agr* abolished expression of RNAIII at all time points tested. Surprisingly, deletion of *saeRS* resulted in increased RNAIII expression at both 3 (1.5 fold, p<0.01) and 6 (2 fold, p<0.001) hours growth compared with strain 923.

#### SaeR

Expression of *saeR* peaked at 3 hours in strain 923 and decreased thereafter (Figure 1A). As expected, expression of *saeR* was abolished in 923*Δsae*. Deletion of *agr* resulted in unchanged expression of *saeR* expression compared with strain 923 at 3 hours, but a modest decrease at 6 hours (2 fold, p<0.05).

#### α-hemolysin (*hla*)

Expression of *hla* peaked at 6 hours in strain 923 (Figure 1A). Deletion of *agr* resulted in unchanged expression of *hla*, compared with 923, at 3 hours (p = 0.13), but decreased expression at 6 hours (3.3 fold, p<0.001). In contrast, an *hla* transcript was undetectable at any time point after deletion of *saeRS*.

#### PVL (lukF-PV)

For strain 923, expression of *lukF-PV* peaked at 3 hrs and decreased thereafter (Figure 1A). Deletion of *agr* resulted in diminished levels of the *lukF-PV* transcript, compared with 923, at 3 (10 fold, p<0.001) and 6 hours (1.8 fold, p = 0.02). Deletion of *saeRS* also decreased *lukF-PV* transcription, compared with 923, at 3 (27 fold, p<0.001) and 6 hours (7 fold, p<0.01).

#### Protein A (*Spa*)

For strain 923, expression of *spa* peaked early and decreased thereafter, as expected (Figure 1A). Deletion of *agr* resulted in markedly increased *spa* expression at 3 (>500 fold, p<0.01) and 6 hours (>3000 fold, p<0.001), compared with strain 923. In contrast, deletion of *saeRS* resulted in no change in the level of *spa* transcript compared with strain 923 at either time point.

### Deletion of *agr* or *saeRS* altered abundance of selected proteins in culture supernatants

#### Hla

As assessed by western blot, Hla abundance increased over time in strain 923 (Figure 1B). It was, however, undetectable in culture supernatants of either 923*Δagr* or 923*Δsae* at any of the time points assessed.

#### Spa

As assessed by western blot, protein A was detectable in culture supernatants of strain 923 at all time points assessed (Figure 1B). Deletion of *saeRS* resulted in less protein A at 2 hrs (compared with 923), but no difference at the other time points assessed. In contrast, there was considerably more protein A in culture supernatants of 923*Δagr* at all time points, consistent with the increased transcription of *spa* observed by qRT-PCR.

### *Agr* and *saeRS* were important for virulence in necrotizing pneumonia

#### Clinical features and mortality

**Figure 2:**
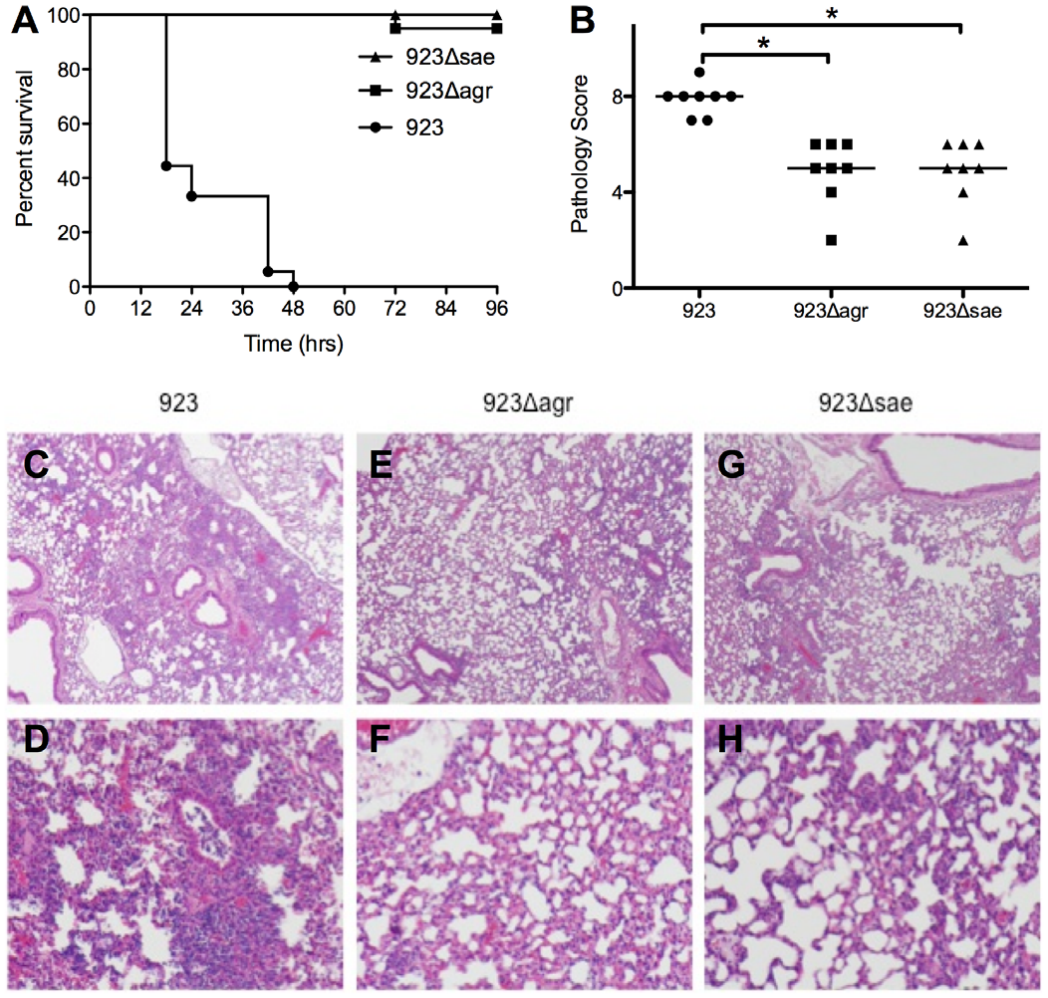
Deletion of *agr* or *saeRS* decreased mortality in a mouse model of necrotizing pneumonia. (A) Mortality after infection with 3×10^8^ CFU *S. aureus* strains 923, 923*Δagr*, or 923*Δsae* (n = 15–20 animals/group). (B) Pathology severity score of lungs of animals assessed 6 hrs after infection with *S. aureus* (n = 8 animals/group). * indicates p<0.05. (C–H) Hematoxylin and eosin stained lung sections examined at low (C–G) and high (D–H) power demonstrate more severe inflammation in the lungs of animals infected with strain 923 compared with strains 923*Δagr* and 923*Δsae*.

Regardless of the infecting strain (923, 923*Δagr*, or 923*Δsae*), all inoculated animals (n = 15–20/group) appeared ill within 6 hrs of inoculation, as characterized by hunched posture, ruffled fur, labored breathing, and decreased mobility. However, by 24 hrs after inoculation, animals inoculated with either 923*Δagr* or 923*Δsae* had recovered and appeared normal. In contrast, more than 60% of mice inoculated with strain 923 died prior to 24 hrs after inoculation, and surviving animals continued to appear ill (Figure 2A). By 48 hrs, all mice inoculated with strain 923 had died, but mice inoculated with 923*Δagr* or 923*Δsae* appeared well. One mouse died 72 hrs after infection with 923*Δagr*.

#### Histopathology

6 hrs after infection with the high inoculum (3×10^8^ CFU, n = 8 animals/group) of *S. aureus*, there was histopathologic evidence of inflammation present in the lungs of all inoculated animals, regardless of the infecting strain. The inflammation was most severe in the lungs of 923-inoculated animals, as reflected by higher pathologic severity scores compared with recipients of 923*Δagr* (p<0.001) or 923*Δsae* (p<0.001) (Figure 2B). Lungs of animals infected with 923 had a wider distribution of inflammation, more severe neutrophil accumulation, and larger inflammatory lesions (Figure 2C-H). Necrosis was not observed in any animals, consistent with previous data showing that necrosis is not visible until at least 9 hrs after infection [17].

#### *In vivo* bacterial survival in the lung

**Figure 3:**
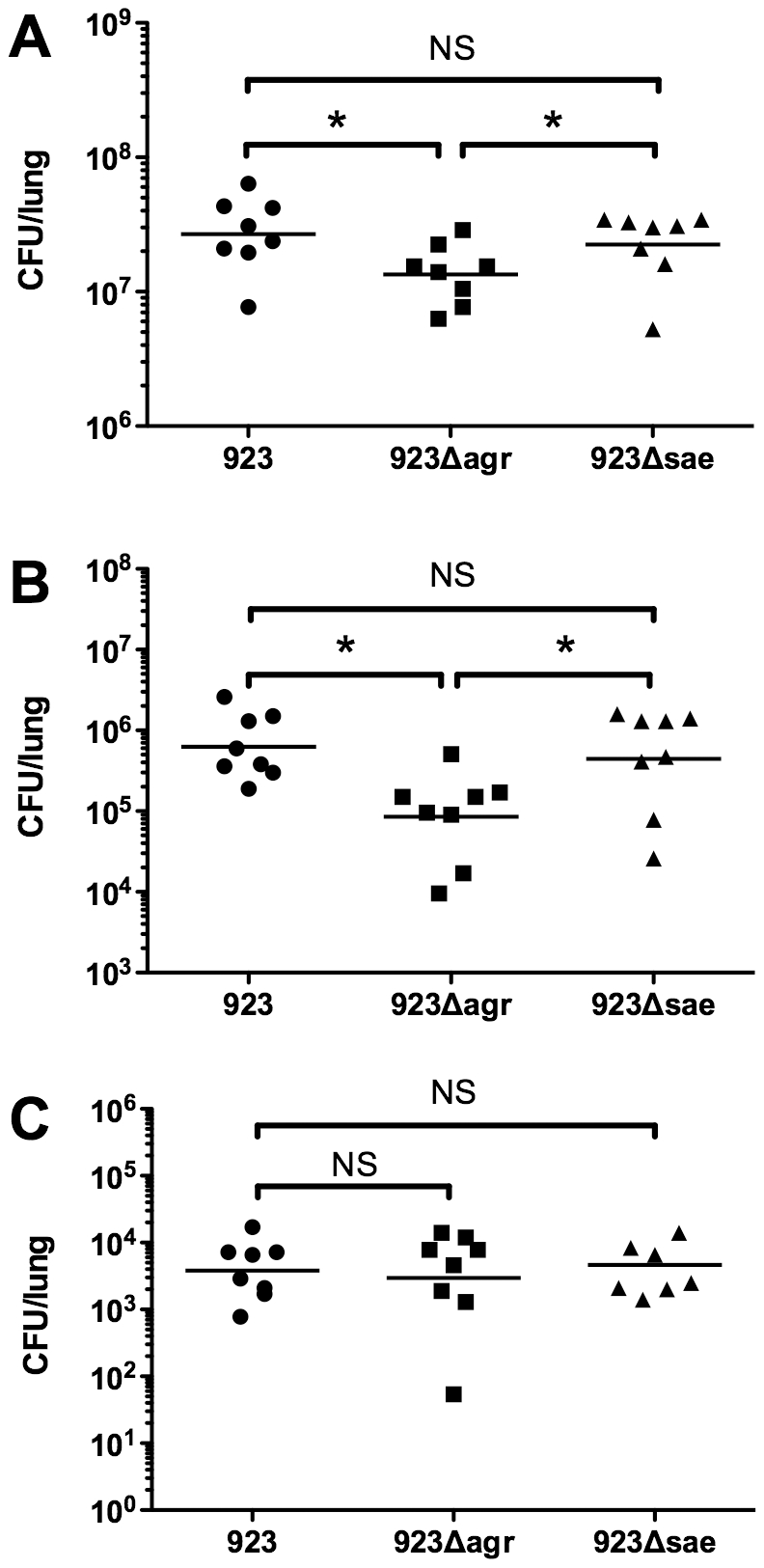
Deletion of *agr* or *saeRS* had modest effects on recovery of bacteria from the lungs in the pneumonia model. In vivo bacterial recovery from the lungs of animals assessed 6 hrs after infection with a high inoculum (3×10^8^ CFU) (A), 6 hrs after infection with a low inoculum (1×10^8^ CFU) (B), or 24 hrs after infection with the low inoculum (C) of *S. aureus* strains 923, 923*Δagr*, or 923*Δsae* (n = 8 animals/group). * indicates p<0.05; NS indicates not significant.

6 hrs after infection with the high inoculum of 923*Δsae*, there was no difference in bacterial recovery from the lung, compared with animals infected with strain 923 (p = 0.43) (Figure 3A). In contrast, there was a modest decrease in recovery of viable *S. aureus* from the lung after infection with 923*Δagr*, compared with either 923 (p = 0.03) or 923*Δsae* (p = 0.04). These results were confirmed 6 hrs after infection with a lower inoculum (1×10^8^ CFU, n = 8 animals/group) (Figure 3B). However, there was no difference among animals infected with 923, 923*Δagr*, or 923*Δsae* in bacterial recovery from the lung 24 hrs after infection with the low inoculum (Figure 3C). Because most animals infected with the high inoculum of 923 died by 24 hrs, we were unable to assess bacterial density in the lungs at the later time point in this group.

### *Agr* and *saeRS* were important for virulence in skin infection

#### Clinical features

**Figure 4:**
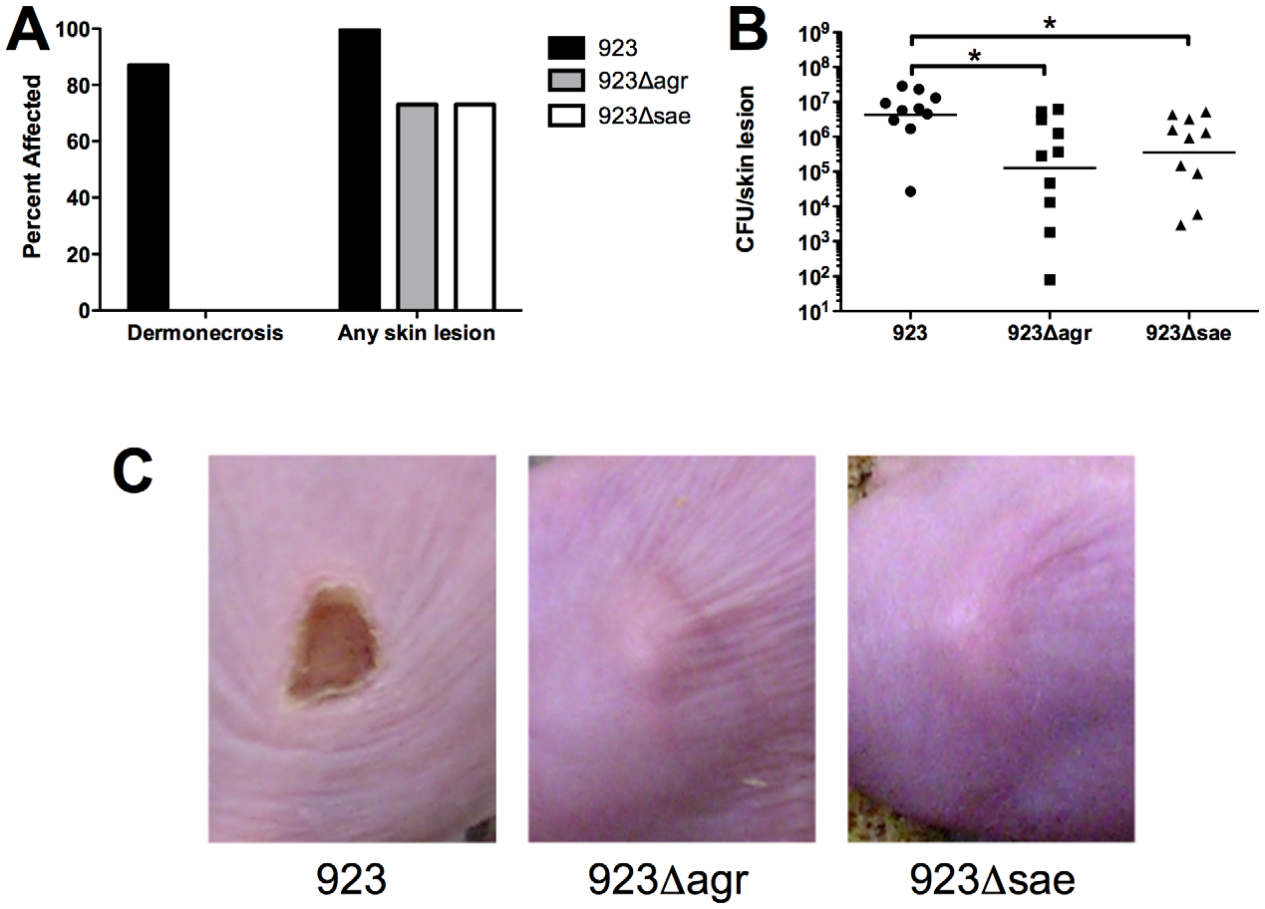
Deletion of *agr* or *saeRS* decreased virulence in a mouse model of skin infection. Mice were infected with 1×10^7^ CFU of *S. aureus* strains 923, 923*Δagr*, or 923*Δsae* (n = 10–20 animals/group). (A) Deletion of *agr* or *saeRS* eliminated dermonecrosis, although abscesses still occured. (B) Deletion of *agr* or *saeRS* resulted in enhanced bacterial clearance from skin lesions 3 days after inoculation. (C) Representative lesions 3 days after inoculation.

Dermonecrotic skin lesions appeared within 24 hrs after infection in 13/15 mice inoculated with strain 923 (Figure 4A, C). Consistent with previous observations, lesions were largest in the 1–2 days after inoculation and decreased in size thereafter (data not shown) [12]. In contrast, there was no dermonecrosis observed in mice infected with either 923*Δagr* or 923*Δsae*. However, abscesses without dermonecrosis were present within 5–7 days in 11/15 mice infected with 923*Δagr* and in 11/15 mice infected with 923*Δsae* (Figure 4A, C).

#### Bacterial recovery from skin lesions

3 days after infection with strain 923, 10^6^–10^8^ CFU were recovered from skin lesions (Figure 4B). The number of bacteria recovered from lesions of mice infected with either deletion mutant was significantly decreased; there was a nearly 10-fold decrease after infection with 923*Δsae* (p = 0.02) or 923*Δagr* (p = 0.02).

## Discussion

The remarkable virulence of USA300 in murine models of infection has been demonstrated by several groups, but the mechanism of this hypervirulent phenotype has remained undefined. The observation that USA300 strains have increased in vitro expression (compared with USA400) of the global regulatory systems *saeRS* and *agr* [7] led us to investigate the possibility that increased transcription of these operons underlies this virulence. Our findings confirm that *saeRS* and *agr* are each important in the pathogenesis of USA300 infections and support a major role for each in the observed phenotype.

*SaeRS* was necessary for mortality in the necrotizing pneumonia model and for dermonecrosis in the skin infection model. This essential role for *saeRS* in pneumonia has not been reported. Our findings in the skin infection model confirm those of Nygaard et al [34]. They found that deletion of *saeR* and *saeS* in LAC, a USA300 isolate, resulted in absent dermonecrosis and decreased bacterial density in lesions 4 days after infection. However, deletion of *saeRS* in MW2, a USA400 isolate, did not affect virulence in a skin infection model [33]. In that model, MW2 caused abscesses without dermonecrosis, which, given the importance of α-hemolysin in dermonecrosis, was likely due to relatively low *hla* expression previously observed in USA400 isolates [7], [21]. Thus, it is likely that the different effect of *saeRS* deletion represents a background-dependent phenomenon; i.e. the greater effect of *saeRS* deletion in USA300 strains was due to increased transcription of *saeRS* (and *hla*) in USA300 compared with USA400 strains [7]. Deletion of *saeRS* also decreased mortality in a mouse model of bacteremia in both the USA300 and USA400 backgrounds [33], [34]. Taken together with our findings, these studies point to an important role for *saeRS* in the pathogenesis of CA-MRSA infection caused by USA300 strains.

Like *saeRS*, *agr* was also necessary for mortality in the pneumonia model and for dermonecrosis in the skin infection model. Inactivation of *agr* has been shown to prevent mortality in a mouse model of pneumonia, although these studies were performed with *S. aureus* strain Newman, an MSSA strain from a different genetic background [37]. Another group reported that deletion of *agr* (in the RN6390 background) decreased the incidence of mortality, pneumonia, and bacteremia after intranasal infection of neonatal mice [38]. Interestingly, in that model, infection with the *agr* mutant still resulted in an inflammatory response [38]. Although the virulence of *agr* deletion mutants has not been reported for models of skin infection, inhibition of *agr*-mediated quorum sensing blocked abscess formation in mouse models of *S. aureus* skin infection [39], [40]. *Agr* was also important in experimental models of *S. aureus* arthritis [41] and osteomyelitis [42].

How *agr* and *saeRS* mediate virulence in *S. aureus* remains to be elucidated, but the similar phenotypes of the *agr* and *saeRS* deletion mutants suggests a common downstream virulence factor (or factors) that are controlled by both. Both *saeRS* and *agr* are believed to be important in the expression of multiple virulence genes, including those encoding alpha-hemolysin, gamma-hemolysin, beta-hemolysin, and the Panton-Valentine leukocidin. We assessed the effects of *agr* and *saeRS* on two well studied toxins, Hla and PVL. Hla is important in the pathogenesis of *S. aureus* necrotizing pneumonia [14] and dermonecrosis [21]. The importance of PVL, on the other hand, remains controversial.

We found an important role for both *saeRS* and *agr* in the control of *hla* in USA300. Deletion of *saeRS* essentially abolished *hla* transcription, which was confirmed by the absence of Hla protein in culture supernatants. These results are supported by the work of Xiong et al, who found that deletion of *sae*, compared with *agr*, had a greater effect on *hla* expression in vitro and in a rabbit model of endocarditis [43]. *SaeRS* promotes the expression of of *hla* in USA300 strains via binding of *saeR* to a conserved sequence in the *hla* promoter region [34]. Deletion of *agr*, on the other hand, resulted in a moderate reduction in *hla* transcription, depending on the time point assessed. Interestingly, we found that deletion of *agr* resulted in the absence of Hla protein in culture supernatants. This discrepancy is perhaps explained, since RNAIII, a major effector of *agr* activity, promotes the translation of the *hla* transcript [44]. These observations confirm that alpha-hemolysin production can be controlled at either the transcriptional (by *saeRS*, and, to a lesser extent, by *agr*) or the translational (by *agr*) levels by these global regulators.

Both *agr* and *saeRS* play a key role in the control of *lukSF-PV*, as deletion of each resulted in significantly decreased transcription. The role of PVL in the pathogenesis of CA-MRSA infections remains controversial [13], [14], [15], [16], [17], [18], [19], [45]. Although the epidemiologic association of the genes encoding PVL with CA-MRSA strains is compelling [20], investigation of the role of PVL in rodent models has yielded conflicting results, possibly because of interspecies variability in susceptibility of neutrophils to the toxic effects of PVL [46]. In support of this, Diep et al recently demonstrated an important role for PVL in the pathogenesis of pneumonia in a rabbit model [19].

Protein A (Spa) is thought to be another key virulence determinant in *S. aureus* pneumonia [47]. Deletion of *agr* resulted in markedly increased transcription of *spa* and increased Spa abundance in culture supernatants. This is not surprising, because *agr* is known to repress *spa* transcription [28]. The fact that the *agr* deletion mutant, despite this abundance of Spa, was less virulent in the pneumonia model suggests that Spa, in the absence (or decreased amount) of secreted toxins such as Hla or PVL, is insufficient for the development of highly virulent disease.

Our studies have also provided some insight into the interactions of *saeRS* and *agr*. Deletion of *agr* had little or no effect on the transcription of *saeRS* in the USA300 background we studied. However, deletion of *saeRS* resulted in increased transcription of RNAIII. Interestingly, despite increased transcription of RNAIII in the *saeRS* mutant, α-hemolysin was not detected in culture supernatants, indicating that RNAIII alone is not sufficient for Hla production. The mechanism by which *saeRS* alters RNAIII transcription is unknown, but similar effects of *saeRS* on *agr* have been shown previously in another *S. aureus* background [48].

We found very little difference in the clearance of bacteria from the lungs 6 hrs after infection and no difference at 24 hrs, regardless of the infecting strain. Moreover, although the severity of histopathology was decreased after infection with either deletion mutant, the differences were relatively modest. There are several possible explanations. Previous work has shown that the characteristic pathologic findings of necrotizing pneumonia, including bacterial replication, are not typically observed until at least 9 hrs after infection [17]. Therefore, it may be that the small differences observed 6 hours after infection would be more pronounced if histopathology and in vivo bacterial survival were assessed later. However, we did not find differences in bacterial clearance 24 hrs after infection. It should be noted that we were unable to assess bacterial clearance 24 hrs after infection with the high inoculum, because most animals inoculated with strain 923 had died by this time. Alternatively, deletion of either *saeRS* or *agr* might result in an altered inflammatory response that determines the outcome of infection (i.e. survival vs. death). Ongoing studies will explore this possibility.

In summary, these studies confirm that the hypervirulent phenotype of USA300 is dependent on increased expression of the global regulators *agr* and *saeRS*, with resultant increased expression of downstream virulence genes such as *hla* and *lukSF-PV*. This work raises several important questions. First, how do *agr* and *saeRS* mediate virulence? Although it seems likely that the effects of *agr* and *saeRS* deletion on virulence are due to the reduced expression of secreted toxins, this study does not directly address the roles of *hla* and *lukSF-PV*. Second, what is the mechanism of such altered expression? One possibility is that there is another regulator upstream of both *agr* and *saeRS* in USA300 strains that is increasing their expression. Another possibility is the loss of inhibition by another upstream regulator, such as *rot* or *sarT*. The mechanisms leading to death in necrotizing pneumonia also remain elusive. Finally, how *S. aureus* (or bacterial components such as Hla, PVL, and Spa) interacts with the host inflammatory response leading to the pathology observed is unknown.
